# The Current State of Cephalopod Science and Perspectives on the Most Critical Challenges Ahead From Three Early-Career Researchers

**DOI:** 10.3389/fphys.2018.00700

**Published:** 2018-06-06

**Authors:** Caitlin E. O’Brien, Katina Roumbedakis, Inger E. Winkelmann

**Affiliations:** ^1^Normandie Univ., UNICAEN, Rennes 1 Univ., UR1, CNRS, UMR 6552 ETHOS, Caen, France; ^2^Association for Cephalopod Research – CephRes, Naples, Italy; ^3^Dipartimento di Scienze e Tecnologie, Università degli Studi del Sannio, Benevento, Italy; ^4^Section for Evolutionary Genomics, Natural History Museum of Denmark, University of Copenhagen, Copenhagen, Denmark

**Keywords:** aquaculture, behavior, cephalopod, cognition, climate change, genetics, neurobiology, welfare

## Abstract

Here, three researchers who have recently embarked on careers in cephalopod biology discuss the current state of the field and offer their hopes for the future. Seven major topics are explored: genetics, aquaculture, climate change, welfare, behavior, cognition, and neurobiology. Recent developments in each of these fields are reviewed and the potential of emerging technologies to address specific gaps in knowledge about cephalopods are discussed. Throughout, the authors highlight specific challenges that merit particular focus in the near-term. This review and prospectus is also intended to suggest some concrete near-term goals to cephalopod researchers and inspire those working outside the field to consider the revelatory potential of these remarkable creatures.

## General Introduction

Cephalopods have long haunted the human imagination as monsters, inspiring mythology dating back to ancient Greek culture (e.g., the Hydra from the labors of Hercules, see Cousteau and Diolé, 1973, p. 72–73, 75; the Gordon Medusa in Wilk, 2000), to legends of sea monsters in Nordic culture and among sailors throughout the middle ages (Salvador and Tomotani, 2014), to the science fiction of the modern world (e.g., Sphere: Crichton, 1988; 20,000 Leagues Under the Sea: Verne, 1988), where they – or creatures strongly resembling them – often lurk in outer space as alien creatures from other worlds (as in the motion pictures *Arrival*^1^ and *Life*^2^, to mention some). And while they were once reviled as “stupid” by Aristotle (1910), and dangerous, as in *Toilers of the Sea* (Hugo, 2002), this unique molluscan taxon has now come to be admired by both scientists, artists and the general public alike (Nakajima et al., 2018). Their growing popularity is reflected in the choice of many aquariums to house them as star attractions, despite the sometimes formidable challenges associated with keeping them. They are also depicted fondly in contemporary culture from computer generated animations in blockbuster films (e.g., *Pirates of the Caribbean; At World’s End*^3^, *Finding Dory*^4^), to clothing, jewelery and artwork, to the surfeit of online videos^5^ featuring cephalopods. Few other invertebrates garner this degree of recognition or status.

**FIGURE 1:**
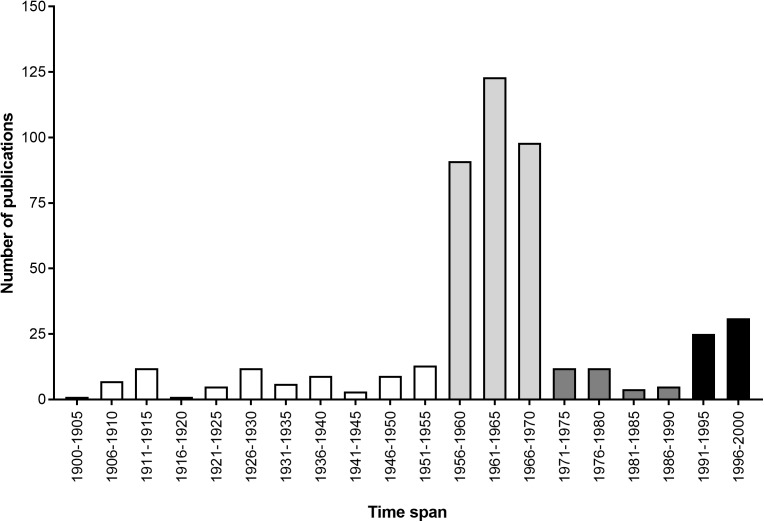
Total number of publications on cephalopods per quinquennium that appeared in a genus-name search of the Zoological Record during the 20th century (adapted from Borrelli and Fiorito, 2008). Bar colors highlight different paces of research (see text for details).

**FIGURE 2:**
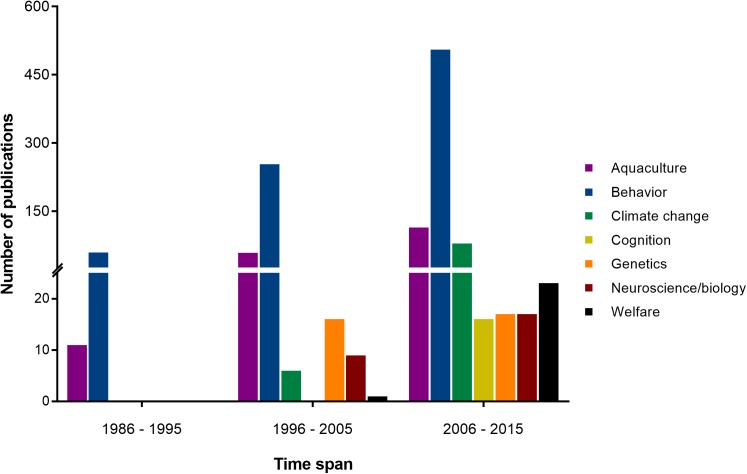
The number of publications per decade between 1986 and 2015 as derived from a search on the Clarivate Web of Knowledge Core Collection (WoS) with “cephalopod” and the research topics addressed in this manuscript used as keywords, i.e., “aquaculture,” “behavior,” “climate change,” “cognition,” “genetics,” “neuroscience/biology,” and “welfare.” Note that total numbers differ between **Figures 1**, **2** due to variations in indexing of the two databases and differences in search criteria.

Cephalopods have also come to be respected for their various contributions to scientific research. During the first half of the 20th century (white bars in **Figure 1**), they played a pivotal role in our understanding of the neuron, thanks to the relative accessibility of the giant axon in squid (Keynes, 2005). This was followed by a period of intense investigation of the cephalopodan nervous system and learning abilities, led by John Z. Young and his fellows, including B. B. Boycott and M. J. Wells among others, from the 1950s to 1970s (see light gray bars in **Figure 1**). Progress slowed from the 1970s to 1990 (see dark gray bars in **Figure 1**), due mainly to a lack of appropriate investigative tools to address outstanding questions (see Bitterman, 1975; see also Young, 1985). Thankfully, the end of the 20th century to the present day has seen a steadily growing body of work concerned with various other aspects of cephalopod biology, including genetics, welfare and the effects of climate change (see black bars in **Figure 1**, and detailed subject-by-subject breakdown in **Figure 2**).

Today, some of the most unique characteristics of cephalopods are also inspiring various technological developments, including adaptive camouflage based on cephalopod skin that can display a variety of patterns (Wang Q. et al., 2014; Yu et al., 2014) or spontaneously match its surroundings (Pikul et al., 2017), suction cups for wound repair (Choi et al., 2016), propulsion and buoyancy systems for Autonomous Underwater Vehicles (AUV, Song et al., 2016), distributed cognitive control systems for artificial intelligence (Íñiguez, 2017) and the design of soft robots (Laschi et al., 2012; Renda et al., 2012).

Despite their great popularity and scientific relevance, detailed information on the biology, ecology, and physiology exists for about 8% (60 species) of the 800 or so known extant species of cephalopods (Jereb and Roper, 2005, 2010; Norman et al., 2014). Much more work is needed if we are to take advantage of all the scientific, technological and cultural inspiration that cephalopods have to offer. In order to stimulate further progress, we here focus on the potential of emerging technologies and of growing interest in cephalopods to address gaps in knowledge in seven particular subfields. We highlight some recent examples of progress in the fields of cephalopod genetics, aquaculture, climate change, welfare, behavior, cognition and neurobiology, and suggest challenges meriting particular focus in the near future (summarized in **Table 1**). The authors are three researchers who recently completed Ph.Ds in cephalopod biology, and who are thus particularly well-positioned (and motivated) to speculate about the future of the field. This manuscript follows from a series of keynote lectures (“Cephalopod Research; Visions of the Future”) delivered during the CephsInAction and CIAC Meeting: Cephalopod Science from Biology to Welfare, held at the CRETAquarium (Crete, Greece, March 28–30, 2017). Hereafter, we first review the current state of cephalopod genetics (an especially fertile area of potential growth) and discuss some of the many ways omic technology can be applied to cephalopod research, including aquaculture. Next, we explore three topics related to cephalopod-human interactions: aquaculture, climate change and anthropogenic impact and welfare of animals in captivity. Finally, we discuss research concerning cephalopod behavior, cognition, and neurobiology, three distinctive biological innovations that occurred during the evolution of this lineage.

**Table 1:** Summary of the most pressing future tasks ahead in cephalopod research as viewed by the authors.

**Genetics**
• Improved phylogenies
• Refinement of eDNA technology
• Genome assembly
**Aquaculture**
• Sustainable food sources
• Control of reproduction in captivity
• Improved healthcare
**Climate Change**
• Determination of thermal tolerances
• Investigation of compound effects
• Particular vigilance for ELS and polar species
**Welfare**
• Validated anesthetics and analgesics
• Non-invasive health and welfare assessment
• Environmental enrichment
**Behavior**
• Field data and naturalistic experiments
• Investigation of inter-individual differences
• Ecotoxicology
**Cognition**
• Use as comparative model
• More precise lineage history
• More information from paleontological record
**Neurobiology**
• Primary neuronal cell culture
• Non-invasive neurological assays
• Brain atlases
**General**
• Open access platform
• Citizen science
• Cephalopod-specific initiatives


**The section on “Aquaculture” was compiled in part from Vidal et al. (2014), Villanueva et al. (2014) and Xavier et al. (2015). The section on “Welfare” was compiled in part from Andrews et al. (2013) and Fiorito et al. (2015).**

## Cephalopods and Genetics

### Current Affairs: Ongoing and Recent Developments in Cephalopod Genetics

The incorporation of genetic tools in cephalopod research has progressed at a relatively slow pace in comparison with other taxonomic groups, as was recently noted by Xavier et al. (2015), and has faced many challenges, such as large and highly repetitive genomes. However, the tide is changing and even in the short time since this previous review by Xavier et al. (2015), there have been several important developments. Generally, DNA sequencing prices have continued to drop per bp sequenced, and the output capacity of commercial platforms has continued to increase, to the point where we can find ourselves inundated with data. Indeed, it is predicted that we will soon be dealing with a field where sufficient data storage and bioinformatic processing resources will be of much greater concern than generation of sequence data itself (Stephens et al., 2015).

Several cephalopod genome projects are in the works, and have been for some years, but the completion and publication of these has been delayed by the overwhelming complexity of cephalopod genomes. Currently running projects include several of those cephalopod species selected by the CephSeq Consortium (Albertin et al., 2012), such as the pygmy squid (*Idiosepius paradoxus*), the bobtail squid (*Euprymna scolopes*), the blue-ringed octopus (*Hapalochlaena maculosa*) and the deep-sea giant squid (*Architeuthis dux*). This initial choice of species^6^ was based on the potential practical use of the animals in a laboratory setting, as well as on particularly interesting and unique biological traits.

As a result of these efforts, a huge milestone was recently reached, when the first cephalopod genome – that of the California two-spot octopus (*Octopus bimaculoides*) – was finally completed and published (Albertin et al., 2015), making front page news in the journal Nature. The main findings were both surprising and fascinating. There was no apparent evidence of a whole genome duplication, which had been previously thought to explain the large genome size and pervasive repeats. The octopus genome was found, instead, to be broadly similar to those of other invertebrates, apart from an immense expansion of two specific gene families, which were previously known to be expanded in vertebrate genomes only. The first of these is the Protocadherins (a type of cell-adhesion proteins), which are particularly important for neuronal development. The second is the C2H2 class of zinc finger transcription factors (small protein structures, which typically function as interaction modules between DNA, RNA, proteins, or other small, useful molecules within a cell), hundreds of which were unique to the octopus. Moreover, these transcription factors were found to be selectively expressed in exactly the kinds of tissues that are special to the cephalopods, such as their suckers, nervous system and color-changing skin. Overall, what this first cephalopod genome revealed is that the expansion and diversification of these two gene families may have played a pivotal role in the evolution of those neural and morphological traits that make cephalopods so exceptional.

### Mind the Gaps: The Problem of Assembling Cephalopod Genomes

As mentioned above, the cephalopods have presented a particular challenge to researchers in terms of assembling their nuclear genomes, in part due to their large size, but especially due to the rampant repetitive regions (strings of the same DNA sequence over and over again) scattered across them (Albertin et al., 2012). The reason this has presented such a problem is to do with the underlying technology of the sequencing platforms which have thus far been commercially available for use. Popular sequencing platforms, like the Illumina HiSeq, require the genomic DNA to be broken into short fragments of a few hundred base-pairs, so that they can be read by the sequencer. These short reads are then assembled, often by the billions, a bit like a large jigsaw puzzle, to re-create the original genomic sequence. This is accomplished with powerful, highly specialized bioinformatic software, such as Meraculous (Chapman et al., 2011).

It is well known that these short-read technologies have limitations for “*de novo*” genome assembly, that is putting together a genome from scratch without prior knowledge or references, when it comes to repetitive regions. The problem arises when the length of the reads from the sequencer is shorter than those repetitive genomic regions that are to be assembled. Picture a gigantic puzzle, made up of tiny square pieces, where many regions of the image are exact copies of each other. How would you work out which copy each piece originally belonged to? It’s an impossible task, and the result has been that *de novo* assemblies of repeat-rich genomes, which have been sequenced with short-read technologies, come out with many gaps and missing parts (Alkan et al., 2010). That is, if they can be assembled at all.

These kinds of problems, however, are (hopefully) about to become a thing of the past. Emerging long-read technologies, such as Pacific Biosciences (PacBio) (Rhoads and Au, 2015), and the Oxford Nanopore (Jain et al., 2016) series, are already available to researchers. These new sequencing technologies can currently produce reads that in some cases are more than a hundred thousand base pairs long, thus overcoming most issues with assembling repetitive regions (Pennisi, 2017). It is still early days, and both of these platforms remain relatively expensive to use and they suffer from higher error rates than the Illumina short-read technology (currently, roughly 15%, compared to only 1% for the Illumina), but this is bound to change, just as it did for the platforms that came before them, and perhaps just as rapidly. Together with improved software algorithms and other clever innovations (a couple of examples are given in Korbel and Lee, 2013; Kitzman, 2016), these developments have led to a recent flood of high-quality plant and animal genomes. This is important, because genome quality makes a big difference for the quality of science it is possible for researchers to do, and it will not be long before the trend includes cephalopods too.

### Seeing the Forest for the Trees: The Importance of Improved Phylogenies

There will be many scientific benefits of this influx of high quality genomes to various fields of cephalopod research. The first of these will be the procurement of more accurate phylogenies. Due to the evolutionary history of the modern coleoid cephalopods, with a rapid radiation of the many different groups happening over a hundred million years ago, combined with their characteristic soft bodies leaving very few fossils, it has been difficult to accurately reconstruct their deep-level relationships to each other using phylogenetic analyses. Mitochondrial genome (Strugnell et al., 2017) and nuclear transcriptome (a genomic approach of sequencing all protein coding genes via their transcribed RNA, Lindgren and Anderson, 2017; Tanner et al., 2017) studies have already made progress toward solving this issue. In doing so, they overturned several previous notions about cephalopod relationships, such as the assumed monophyly of the squids, but with different published datasets also recovering slightly different phylogenetic trees. A couple of factors that have been found to influence the topology are marker coverage, that is how much of the genome is available for comparison in all of the sequenced species, and taxon sampling, that is how broadly and densely species were sampled across the true phylogeny (Lindgren and Anderson, 2017). High quality genomes from a growing number of cephalopod species will help to amend these problems, and, hopefully, finally provide a resolved picture of their evolutionary history.

The availability of accurate phylogenetic trees is crucial for studies of comparative evolutionary biology, as they allow independently observed traits to be mapped onto them, revealing the evolutionary histories of these traits and helping researchers to distinguish between functional similarity and relatedness. Some traits may be shared because several species share an ancestor who carried that trait, while other traits may be shared between species due to convergent evolution – the independent invention of the same functional trait more than once. This ability to unravel the history of morphological or behavioral trait evolution, and to classify traits as ancestral or derived, is highly relevant, for instance, to the study of cephalopod neurobiology and cognition, as well as other cephalopod specializations such as evolution of the ink sac, vision, acquisition of symbionts and toxin production.

### Plastic Fantastic: A New Model for Fundamental Research on Genome Plasticity

Yet another intriguing specialization of the coleoid cephalopods, which we have only just begun to discover as we probe their genomes, is prolific RNA editing. While only a handful of a human’s roughly 20,000 genes yield edited RNA transcripts (Pinto et al., 2014), a recent study found that more than half of translated gene transcripts in coleoids are edited, making it the rule rather than the exception (Liscovitch-Brauer et al., 2017). This pattern was not found in their distant relatives, the nautiloids, or in other molluscs. Moreover, most of these edits (65%) were found to change the amino acid sequence in the resulting protein, and are thus meaningful to the development of the animals. In neural tissue of *O. bimaculoides* specifically, 11–13% of edits change the amino acid, compared with less than a percent in mammals. Interestingly, many of these changes were made in the Protocadherins, that same gene family found to be massively expanded in the octopus genome. This implies that the behaviorally complex coleoid cephalopods have invented another ingenious way to quickly change and diversify the expression of their genome, especially in the genes important for their neural development. The extensive RNA editing to diversify their neural proteome does, however, appear to come at the cost of limiting their genomic DNA sequence flexibility and evolution. The flanking regions of the genes, which are important for the editing enzymes to perform their task, and which make up more than a quarter of the entire exome (the protein coding parts of the genome), are highly conserved, and seem to be evolving more slowly than in other animals.

This phenomenon is unparalleled in any studied vertebrate. It is yet another example of cephalopods taking a, sometimes strikingly, different evolutionary route to solve a similar problem, just as they have done for complex eye development (Nilsson, 1996), multidimensional vision (Temple et al., 2012; Stubbs and Stubbs, 2016), and fast action potential velocity in their giant axons versus our own myelin-insulated axons (Hartline and Colman, 2007). For this reason, coleoid cephalopods are likely to become the future model for studying RNA editing and genome plasticity, just as they became the first model for the experimental study of neuronal function after the discovery of their giant axon.

### Fast Forward Selection: The Potential of Genomic Tools for Cephalopod Aquaculture

Another important development, which will be relevant for staking out future directions in genetic work, is a recently revived interest in the culture of cephalopods for experimental purposes, for ornamental aquarium trade, and for commercial food production. Currently, only small-scale culture is possible, and just for a small handful of species (Vidal et al., 2014; Xavier et al., 2015), but the intensity of research into husbandry techniques is increasing, and is likely to result in significant improvements over the coming years. This means that researchers may very soon unlock the potential of cephalopods to be kept and studied as an experimental laboratory model organism, much like mice, only with many traits that are extraordinarily similar to those of vertebrates, yet with an independent evolutionary history. It will also be important for industrial-scale aquaculture, as the world’s wild cephalopod stocks are under increasing pressure as a fisheries resource (Rodhouse et al., 2014).

When it comes to keeping cephalopods as cultured animals, just learning how to farm the wild-type cephalopods, as they occur naturally, is unlikely to be enough for efficient results. Just as it has been the case for domesticated animals and plants in the past, it is likely to be in the future; humans have proven highly skilled at selecting and shaping our chosen creatures. Over the centuries, humans have domesticated animals for meat, dairy and company (Wang G.D. et al., 2014), plants for food, decoration and raw materials (Meyer and Purugganan, 2013), as well as microorganisms for food fermentation (Douglas and Klaenhammer, 2010). Unlike these past events, however, contemporary domestication of animals and plants comes with an unprecedented set of tools for breeders, such as genomics. With genomic information and information about the heritability of phenotypic traits, suitable technologies for marker-assisted selection, genome selection, and genome editing can be developed for applications in aquaculture.

High quality genome resources make genotyping of individual animals easy, and eventually cheap, for breeders. Genomic selection has already been a huge success in dairy cattle breeding, which since 2009 has not used progeny testing as a standard for evaluating young bulls, instead relying purely on genomic information (Boichard et al., 2016). The techniques first became common practice at a large scale in the most common breeds, but as prices dropped and efficiency rose, they quickly became a useful tool for less common cattle breeds as well.

Traits of interest for genomic characterisation, heritability assessment and selection in cephalopods for aquaculture include, but are not limited to, feeding preferences, environmental stress and crowding tolerance, disease resistance, size, growth rate and timing of sexual maturation. Much progress has already been made in aquaculture genomics for dozens of fish and shellfish species, including various stages of genome reference sequences, the development of genetic linkage maps, single nucleotide polymorphism (SNP) chip arrays and transcriptome databases (Abdelrahman et al., 2017). With the expansion of available genome resources, which is sure to follow further drops in sequencing prices and implementation of new sequencing technologies, and the substantial economic interest in large scale culture of cephalopods for consumption, this group of animals is sure to follow.

Given the era in which cephalopod domestication will happen, aquaculturists will not be limited only to the genomic technologies available to breed developers today. New and exciting possibilities lay ahead, such as the constantly, and rapidly, improving DNA base-editing technology (and also RNA, see Cox et al., 2017) derived from the molecular scalpel CRISPR-Cas9 (Gaudelli et al., 2017). CRISPR (Clustered Regular Interspaced Short Palindromic Repeats) is part of a rudimentary microbial adaptive immune system, which was only discovered in the 1990’s and first understood in 2005 (Mojica et al., 2005). There is debate about which research group broke first ground in harnessing the system for genome editing around 2012, but while that debate continues, researchers across the globe are testing and refining the technology at a dizzying pace.

The development of such precise and easily programmable gene editors has enormous implications for quickly engineering high-performance aquaculture breeds. A case in which successful gene-editing has already been performed to attain a highly desired phenotype for a commonly cultured animal is that of the double-muscled pig. Double-muscled animals, such as the cattle breed Belgian Blue, are animals with a massive increase in skeletal muscle mass, which are known to occur naturally among cattle at very low rates (Fiems, 2012). Carriers of the gene cannot be identified by their phenotype, but since the mutation causing the phenotype was discovered (a so-called ‘knock-out’ mutation causing a non-functional myostatin gene), it became instantly possible, first of all, to identify carriers by genotyping. Recently, it also led to the engineering of other species with a similar phenotype, by using gene-editing to knock out that same gene. The first double-muscled pigs (Cyranoski, 2015) were reported to have been engineered by researchers using TALEN (Transcription Activator-Like Effector Nucleases) gene editing, a slightly older technique that has been in use since 2011 and was crowned a Nature Method of the Year that same year (Anon, 2011). The same result could have been accomplished, as it was for the Belgian Blue, with traditional breeding methods, but that did take almost 200 years. Likewise, with sufficient understanding of the genomics of cephalopods, it may be possible to further increase the speed with which we can adapt the animals to aquaculture, by using gene-editing to copy any desired, genetically determined, traits between species or breeds.

### Free-Floating Data: Using Environmental DNA for Next Generation Ecology

Finally, another development in genetics, which holds great potential for cephalopod research, lies in the realm of population ecology and biodiversity monitoring. It is the emerging technology for environmental DNA (eDNA) detection and analysis. With eDNA analysis, DNA is isolated directly from an environmental sample, such as soil or water, without first isolating any type of organism. The technology for working with eDNA has its roots in the field of soil microbiology, where it was initially used to detect DNA from microbial life in sediments, but it has since been successfully adapted for eDNA from a wide range of sources, including sea water (Thomsen et al., 2012). Although detection of animals from eDNA sampling has thus far focused mainly on targeted sets of species or genera, it is anticipated that studies of eDNA will increasingly focus on meta-genomic surveys of entire ecosystems to investigate spatial and temporal patterns of biodiversity (Thomsen and Willerslev, 2015). This could be a tremendously useful technology to apply to the study of cephalopod populations, especially pelagic, because so little is currently known. This is mostly due to the difficulty of finding and observing the animals, and of collecting direct tissue samples. Shallow-water cephalopod species and those that are either commercially important for fisheries or easy to raise in captivity are the most studied and best understood. However, roughly 45% of all known cephalopod species are non-commercially important open-ocean or deep-sea squids and octopods (Sweeney and Roper, 1998). Application of eDNA methods have been shown to work for detecting and monitoring not only common species, but also those that are endangered, invasive, or elusive (Bohmann et al., 2014), and it could therefore be an especially potent tool for presence/absence monitoring of pelagic cephalopod species. Eventually, further development and application of these tools will open up an entirely new avenue for the study of population and community dynamics of these cephalopods.

Because detectable eDNA does not persist in the marine environment for long (Thomsen et al., 2012), the results are in real time, and the methods can be applied on any time-scale, from the monitoring of daily migration patterns to whole population range shifts in response to variations in climate. It can also be adapted from the collection of free floating DNA, using nets with different mesh sizes, to target single cells or planktonic organisms of a specific size, such as elusive cephalopod paralarvae. Furthermore, it may not remain restricted only to presence/absence or relative abundance estimates. Remote population genetic analyses may also be possible, as was recently demonstrated for an aggregation of whale sharks (*Rhincodon typus*) in the Arabian Gulf (Sigsgaard et al., 2016; Creer and Seymour, 2017). The authors collected sea water samples totalling 30 L and, incredibly, used them to estimate the genetic diversity of the whale shark population currently occupying the area.

Another exciting technological advance in the realm of eDNA is the so-called Environmental Sample Processor (ESP) developed at MBARI. It is a robotic device, which filters seawater down to 4000 m depth and applies a variety of molecular assays to the water samples, including quantitative PCR (qPCR), to identify specific target organisms and genes *in situ* (Ussler et al., 2013). This means it is not even necessary to collect water samples manually. Instead, the ESP can be placed or buoyed at a survey site, and programmed to test for the presence of defined species or genera at specific time intervals and transmit the results to researchers remotely. For now, the ESP is built with qPCR capabilities, but there is no reason this should not be upgraded to a type of small high-throughput sequencing technology in the future, thereby expanding its capabilities from a restricted focus on the target species (or genus), to a tool which can perform remote surveys of biodiversity and composition of entire biological communities.

Lastly, the impact of eDNA methods may even be felt by researchers working on aquaculture and breeding of cultured cephalopods as well. One of the problems when working with small and vulnerable larvae of cultured organisms is that of collecting sufficient DNA for genotyping without causing lethal injury to the larvae. This problem has recently been solved by genotyping of free-swimming, early fish larvae in a non-lethal and non-invasive way, by collecting and characterizing their eDNA (Espinoza et al., 2017), in a way that could quickly be adapted for cephalopods.

What treasures the future of genetics holds.

## Human Impacts on and Interactions With Cephalopods

### Culturing Consensus: Best Practices for Cephalopod Husbandry

Cephalopods are cultured for a variety of reasons, including human consumption, public display and restocking (Iglesias et al., 2014; Nabhitabhata and Segawa, 2014; Vidal et al., 2014). The potential use of aquacultural by-products, including pharmaceutical compounds (Koueta et al., 2014) is seen as another potential benefit. Certain species are particularly well-suited to aquaculture due to their rapid growth, short life cycles and market value (Lee et al., 1994; Pierce and Portela, 2014). Culture techniques have been developed for some of the species consumed for food, displayed in aquaria or used for scientific purposes, and information regarding capture methods, basic requirements (e.g., water quality, tank systems, hatching conditions, etc.) and diets (e.g., natural and artificial) for these species have been published (Boletzky and Hanlon, 1983; Iglesias et al., 2014; Vidal et al., 2014). There is, however, a consensus that two major obstacles limit large-scale and sustainable cephalopod culture in general: a lack of knowledge regarding optimal nutritional requirements and difficulties associated with successful reproduction in captivity (for details see **Table 1**).

The ultimate goal of cephalopod aquaculturists is the development of sustainable artificial diets, preferably based on non-marine ingredients (less expensive than marine equivalents), or derived from the discarded by-products of other fisheries (Vidal et al., 2014; Villanueva et al., 2014; Xavier et al., 2015). Cephalopods are short-lived and thus fast-growing animals, due to highly efficient ingestion, digestion and assimilation of proteins. They are also active swimmers and predators, consequently exhibiting a high metabolic rate and considerable demand of food (Boyle and Rodhouse, 2008). Understanding the process by which proteins and other nutrients are digested and assimilated can help to better design diets (e.g., Martínez et al., 2014).

A better understanding of digestive physiology and the feeding habits of each life stage is also needed in order to properly tailor diets to the requirements of specific cephalopod species. Formulated diets should be visually attractive and have proper texture and palatability, as well as appropriate digestibility (Villanueva et al., 2014). This tends to be especially difficult for the early planktonic life stages and juveniles of some species (e.g., *Octopus vulgaris*), which are active visual predators with high metabolic activity and sophisticated predatory behaviors (for review see Nande et al., 2017). On the other hand, it may be possible to facilitate changes in prey preferences during rearing, and train cephalopods to feed on artificial diets due to the remarkable behavioral plasticity of many cephalopod species (Vidal et al., 2014; Villanueva et al., 2014).

Knowledge about the processes and timing of digestion in candidate species for aquaculture is also lacking. A better understanding of how external factors (e.g., temperature, light cycle) can influence these processes, as well as their relation with circadian rhythms, is also needed. Due to differences in environmental temperature, species from different geographical regions (e.g., *Octopus maya* and *Octopus mimus*) will vary in digestive dynamics, the temporality of digestion and in their efficiency and patterns in absorbing and assimilating nutrients (Linares et al., 2015; Gallardo et al., 2017). It is also possible that similar differences occur between disparate populations of species with wide geographical distribution (e.g., *Sepia officinali*s, *Sepioteuthis lessoniana*). Additionally, understanding the physiological regulation of appetite, food intake (feeding frequency and amount of food ingested) and digestion will enable the design of feeding protocols and timetables that can maximize growth and survival rates. Such investigations will greatly optimize the growth and survival of these species at each life stage and improve our ability to properly maintain these animals in captivity, likely increasing both productivity and welfare at the same time (Sykes et al., 2017a).

Techniques for the management of cephalopod reproduction must also be improved in order to enhance aquacultural yields and overcome production bottlenecks. Several specific advances are necessary according to recent reviews (Vidal et al., 2014; Villanueva et al., 2014; Xavier et al., 2015): (i) the development of protocols for accelerating and/or retarding sexual maturation and spawning, thus allowing the control of reproduction under laboratory conditions; (ii) a better understanding of the influence of natural variables (e.g., temperature, photoperiod) on sexual maturation, reproductive performance, spawning, embryonic development and hatching success; (iii) the development of methods (hormonal or otherwise) to induce reproductive maturation; (iv) the improvement of broodstock conditioning and a better understanding of maternal effects on hatching quality and offspring competence; (v) greater knowledge of the role of chemical messaging, olfaction and sex pheromones in reproduction and its associated behavior.

Additionally, cephalopods can be subject to maternal effects, due to differences in embryo provisioning, egg placement, maternal care (for octopods and some squid) and stress-induced changes in behavior (Bloor et al., 2013; Juárez et al., 2016; O’Brien et al., 2017), and a better understanding of these may lead to improvements in hatching success and offspring fitness. Likewise, the potential for paternal effects on offspring should also be investigated, as this is known to be an influential factor in other animal groups (for review, see Rando, 2012) but, to our knowledge, has not yet been investigated in cephalopods. Finally, reproduction in cephalopods is further complicated by the existence of polyandry (e.g., Naud et al., 2005; Squires et al., 2012, 2014; Morse et al., 2018), sperm competition (e.g., Hanlon et al., 1999; Wada et al., 2005) and multiple male mating strategies that exist in several species of cephalopods (Hanlon et al., 2005; Iwata et al., 2011). A better understanding of these dynamics could potentially enable higher fertilization rates and reduce the number of injuries related to male-male competition for females.

The majority of the information available about cephalopod brooding behavior, reproduction and their physiological bases has been obtained under laboratory conditions. Unfortunately, certain information can only be derived from fieldwork. For example, the observation that wild female octopuses often repeatedly open and close the entrance to their den in order to facilitate the release of hatchlings (Cosgrove, 1993; Garci et al., 2016), gives cephalopod keepers insights with which they can improve environmental enrichment for captive brooding cephalopods. Providing materials that allow brooding octopuses to perform this behavior in captivity (e.g., by using natural benthic debris rather than plastic or any other artificial material for shelter/den) could improve reproductive outcomes, reducing maternal stress and improving welfare. More field observations and studies (e.g., direct observation of mating, egg-laying and brooding in the wild, larva counts from plankton tows, etc.) would greatly augment our current knowledge and lead to improved reproductive yields as well as better animal welfare.

In addition to improving management of nutrition and reproduction, cephalopod researchers should strive to establish a set of standardized husbandry techniques for commonly cultured species. As with diet, the culture of the same species may require different standards in tropical and temperate regions, and so region-specific guidelines may be required for certain species (e.g., *Sepia officinalis*, whose range extends from the Northern Atlantic and English Channel to the Mediterranean Sea). Particular attention should be given to the development of adequate artificial incubation techniques for small-egged species, such as *O. vulgaris*, which produce small, delicate planktonic paralarvae (Vidal et al., 2014; Villanueva et al., 2014). In the wild, planktonic paralarvae naturally experience very high mortality rates, and in the laboratory, survival rates are reduced further due to a lack of appropriate food sources and standardized culture systems, as well as due to trauma caused by contact with tank walls (Iglesias et al., 2007; Vidal et al., 2014). In general, research that focuses on facilitating life stage and phase transitions will further aquacultural aims, since these are the most critical and vulnerable periods of the life cycle (Vidal et al., 2014). As discussed previously, genetic manipulation may one day provide a means of controlling cephalopod reproductive capacity and success. Genetic selection/manipulation and biased genomic assays targeting potential genes of interest (e.g., those related to broodstock features, control of sexual maturation, growth, immunology and pathology) are potential methods to be employed (Vidal et al., 2014; Xavier et al., 2015). For example, genetic selection might be used to help sustain cultured populations through multiple generations by selecting for traits that improve success during challenging portions of the life cycle (e.g., reproduction, larval settlement) that currently limit aquacultural production (Vidal et al., 2014).

A better understanding of the functioning of the cephalopod immune system, along with its potential pathologies, infections, parasites and diseases, is critical to optimizing aquacultural output and animal welfare. It is well known that poor culture practices in commercial fish farms tend to compromise animal well-being and to encourage the outbreak of disease (Huntingford et al., 2006; Ashley, 2007). The conditions associated with intensive aquaculture (e.g., confinement, overpopulation and stress) tend to facilitate the incubation and transmission of parasites and disease. Parasites and pathogens normally found in wild populations, may, in many cases, also be responsible for diseases in captivity (Lafferty et al., 2015). Thus, knowledge of the pathogenic agents in wild populations of commonly cultured cephalopod species may aid in the prevention of disease outbreaks and the early diagnosis of health problems when they do occur, preventing or minimizing economical losses. The standardization of techniques for the collection, identification and documentation of parasites and pathogens would greatly facilitate this process and allow important information to be shared more easily.

Cephalopod aquaculture may also be advanced using techniques employed with other animal groups, but not yet tested in cephalopods. For instance, in some commercial fish farms, probiotics (microorganisms introduced to a host for its beneficial qualities) are used to promote growth, improve water quality, prevent disease, increase stress tolerance, enhance immune responses, and serve as a supplemental source of nutrients and digestive enzymes (Balcázar et al., 2006; Cruz et al., 2012; Michael et al., 2014). In cephalopods, however, the potential use of probiotics remains completely unexplored. Future work should focus on the identification of the intestinal biota of wild healthy cephalopod species and the identification of potential probiotic strains.

Finally, because most cephalopod aquaculture is focused on a small number of benthic, shallow-water species, almost no information is available for offshore, pelagic and deep-sea cephalopods (Vidal et al., 2014; Xavier et al., 2015). Given recent interest in the aquarium display of such creatures (e.g., the vampire squid), special attention should be given to the refinement of capture and transport methods for these species and to understanding their nutritional, behavioral and environmental requirements. Such knowledge will improve welfare and boost husbandry success, as well as facilitate the uniformity of experiments conducted on these species in disparate locations.

### Forecasting the Future: Cephalopod Research and Climate Change

The effects of global climate change in marine environments include ocean warming, acidification and changes in dissolved oxygen availability. The consequences of these changes on marine organisms are of growing concern. Ocean warming is likely the most relevant of these changes to cephalopods: it may increase growth rates (if enough food and oxygen are available), consequently accelerating their life cycles (Doubleday et al., 2016) and increasing population turnover (Pecl and Jackson, 2008). Moreover, higher temperatures can shorten the length of embryonic development and increase the likelihood of premature hatching, both of which may cause serious biological impairments during crucial early life stages (Repolho et al., 2014; Caamal-Monsreal et al., 2016; Uriarte et al., 2016).

Thermal windows (the temperature range within which an animal performs optimally) differ between life stages in a given species as well as between species (Pörtner and Farrell, 2008). Establishing thermal windows and tolerances (especially critical thermal maxima, CTMax) for important species should be a priority since these biological limits have implications for the reproductive success and survival of juveniles. In particular, studies evaluating the thermal sensitivity and tolerance of embryos and early life stages are essential to better understanding how these animals will respond to a warming environment, since these are believed to be the most vulnerable stages within the life cycle (Rosa et al., 2012). Published aquacultural guidelines may also need to be periodically updated as species adapt to changing conditions. In particular, cephalopod populations residing in the Arctic and Antarctic may be more susceptible to climate change than populations in other regions due to the heightened environmental sensitivity and volatility of the polar regions (e.g., changes in temperature as well as changes in salinity from melting sea ice) and thus should be monitored especially vigilantly (Xavier et al., 2018).

Ocean deoxygenation and eutrophication, phenomena primarily attributed to the effects of ocean warming, also have implications for cephalopods. Marine hypoxia events have been found to alter the depth distribution of certain squids, as seen in *Dosidicus gigas* (Seibel, 2015). The effects of environmental deoxygenation can also be mediated by thermal tolerance to further affect cephalopod physiology: They can experience thermally induced oxygen limitation due to a reduction of the oxygen binding properties of haemocyanin (which is highly temperature-dependent), limiting survival time and eventually causing premature death (Melzner et al., 2007). In addition, physical abnormalities, such as defects in external yolk sac morphology, reduced embryonic size, as well as mantle, eye and arm deformities (potentially caused by a combination of temperature variation and hypoxic conditions during embryonic development) can occur in newly hatched specimens, as observed, for example, in *Sepioteuthis australis* (Gowland et al., 2002; Steer et al., 2002).

Similarly, ocean acidification could have deleterious effects on cephalopods, such as degrading the hard parts of their anatomy, e.g., cuttlebones (Gutowska et al., 2010; Kaplan et al., 2013), statoliths (Kaplan et al., 2013), and the external shells of nautiluses and argonauts (Wolfe et al., 2012), in addition to altering development time and hatching rate (Kaplan et al., 2013; Xavier et al., 2015). Global changes in oceanic currents may also affect the planktonic paralarvae of cephalopods, and the consequences of this may be positive, negative or both depending on the species (Xavier et al., 2015). Potential positive effects include the colonization of new areas and consequent expansion of species range (Zeidberg and Robison, 2007; Golikov et al., 2013), while potential negative effects include changes in food availability and impacts to the transport of early life stages (Pierce et al., 2010).

Although some information regarding the effects of isolated aspects of global climate change on cephalopods exists in the literature, the impact of combined effects (i.e., ocean warming plus acidification and marine hypoxia, etc.) are, to date, poorly known. Furthermore, questions about cephalopod tolerance and adaptability in the face of changing environments abound. One recent study suggests that the plasticity inherent to cephalopods may allow them to adapt more rapidly than other animal groups: coleoid cephalopods exhibit unprecedented levels of post-transcriptional modification to RNA, allowing the diversification of proteomes beyond the genomic blueprint (Liscovitch-Brauer et al., 2017). This ability may enable them to handle the effects of global climate change more rapidly and adeptly than other animal groups, contributing to increases in cephalopod populations that have been observed around the globe (Doubleday et al., 2016). Nevertheless, while cephalopods may benefit in some ways from a changing ocean environment (Doubleday et al., 2016), population dynamics are difficult to predict and human activities may yet have unpredictable deleterious effects. We must remain vigilant for these.

### Improving Welfare: An Ethical Approach to Cephalopod Research

In the last decade, cephalopod welfare has gained much attention. This is due, in large part, to their addition to the list of animals regulated for use in scientific procedures within the European Union (European Parliament and Council of the European Union, 2010; Andrews et al., 2013; Smith et al., 2013; Di Cristina et al., 2015). Directive 2010/63/EU stipulates that all surgical and investigative procedures applied to vertebrates and now also cephalopods for research purposes should be carried out in such a way as to minimize pain, suffering, distress and lasting harm (PSDLH). In accordance with this principle, experimental procedures should be carried out under anesthesia and analgesia whenever possible and when sacrifice is necessary, animals must be killed humanely. Moreover, cephalopods used for scientific purposes must be maintained under conditions which meet basic health and welfare standards, and have their well-being monitored regularly. Here, we discuss the challenges that remain obstacles to fulfilling these mandates.

Around 20 substances and/or combinations of anesthetic agents have been tested in a few cephalopod species with some apparent success (for review, see Gleadall, 2013; Fiorito et al., 2015), but knowledge of their mechanisms of action is very limited. Moreover, descriptions of cephalopod behavior during anesthetic induction and recovery (e.g., Andrews and Tansey, 1981; Gonçalves et al., 2012; Gleadall, 2013; Butler-Struben et al., 2018) or of the physiological effects of putative anesthetic agents on the animals (Pugliese et al., 2016; Butler-Struben et al., 2018) are relatively few. Variations in the effectiveness of anesthetics in relation to cephalopod age, sex, life stage, body weight, physiological condition and health status, remain largely unexplored, as do the interactions of anesthetics with various parameters, such as temperature, salinity, pH and oxygen level. All of these factors are critical for the humane treatment of animals in experimental contexts, and also for husbandry, which may require anesthesia during handling and surgical procedures.

The information available for analgesia in cephalopods is even more limited than for anesthetics (Andrews et al., 2013; Fiorito et al., 2015). Although ketoprofen and butorphanol have been proposed as analgesics for cephalopods, the dosing guidelines are based on studies performed on fish and amphibians (Gunkel and Lewbart, 2008) and, to date, there are no specific studies testing these substances in cephalopods to the best of our knowledge. Tests of potential analgesic agents and evaluation of their effectiveness are urgently required. This would be facilitated by the development of pain scales, such as those proposed for mammals (e.g., Mouse Grimace Scale, Miller and Leach, 2015). In addition, tests of analgesic self-administration for pain relief, such as those utilizing facultative oral administration in mammals (e.g., Colpaert et al., 1980, 2001), could be used to evaluate a substance’s efficacy in cephalopods.

Protocols for the humane killing of cephalopods also require refinement. Although recommendations of methods have been published (Fiorito et al., 2015), no specific guidelines are provided by Directive 2010/63/EU. The suitability of the methods currently in use needs to be validated and alternative methods should be tested. Future studies should also focus on evaluating the level and nature of any suffering caused by these methods. Apart from pain assessment, a standardized way to assess of responsiveness to stimuli (i.e., consciousness) should be developed so that current and proposed methods of humane killing can be evaluated objectively.

Determining how to properly assess health and welfare in cephalopods is a critical issue to address in the near future but developing species-specific guidelines for welfare assessment and ethical treatment is not an easy task. One potential model for cephalopod welfare assessment is a scored model, based on animals’ physiology (e.g., respiration, osmotic balance, nutrition) and behavior (e.g., feeding, rest, sexual behavior), such as the one designed for the Atlantic salmon *Salmo salar* by Stien et al. (2013). Another potential technique is the use of cognitive assays, such as preference tests, to assess animals’ status (Brydges and Braithwaite, 2008). Some efforts in this vein have been made in recent years. A list of potential indicators for health and welfare in cephalopods, utilizing overall appearance, behavior and clinical indicators, including a graded severity scale, has recently been published (for details see Table 5 in Fiorito et al., 2015). In addition, an attempt to develop a framework for monitoring and assessing cephalopod welfare a “Cephalopod Welfare Index” is currently underway^7^ under the aegis of the COST Action FA1301.

The development of non- or minimally invasive methods to assess the health of cephalopods is needed. For instance, ultrasonography is considered to be a suitable tool to determine sex and the maturation status of the gonads, and to assess the body condition of living animals. In *O. vulgaris*, ultrasonography has also been used to observe mantle contractions during locomotion and respiration (Tateno, 1993), the central nervous system (Grimaldi et al., 2007), the arms (Margheri et al., 2011) and the digestive tract (Ponte et al., 2017). In *S. officinalis*, ultrasound has been used to analyze cardiovascular activity (King et al., 2005), as well as cardiac and ventilatory rates in response to sudden visual stimuli (King and Adamo, 2006). While the potential of ultrasound imaging as a non-invasive method for assessing health in cephalopods is clear, further refinement is required, including the establishment of standardized protocols to assess normal (and abnormal) physiological conditions (e.g., assessment of cardiovascular and respiratory function, reproductive status, parasite infection).

In addition to ultrasound, other non- or minimally invasive methods have recently begun to be explored. For instance, a series of techniques, including behavioral responses to prey, the rate of food intake, fluctuations in body weight, oro-anal transit times, defecation frequencies, fecal appearance and composition, endoscopic assays, and needle biopsy (which may require ultrasound guidance) have been suggested as methods to assess the digestive health of cephalopods (Ponte et al., 2017). Another group of researchers have recently tested methods for *in vivo* sex determination of adult cuttlefish (*S. officinalis*) using an endoscope (Sykes et al., 2017b). Additionally, they suggest the use of subcutaneous elastomer implants for marking individuals and of mucus swabs from the inside of the mantle cavity to obtain DNA samples as minimally invasive techniques to be utilized with cephalopods. The extension of these techniques to other species, and the development of other non-invasive approaches may contribute to better *in vivo* assessment of cephalopod health status and assist in future efforts to improve cephalopod welfare in captivity.

The conception of welfare encompasses not only animal maintenance and basic health care, but animals’ “psychological” well-being as well. In addition to having their basic physiological needs met and not suffering from discomfort, pain or stress, cephalopods used as experimental subjects or kept in public aquaria should be free to express their natural behavior (Mellor, 2016). As such, “enrichment” of housing conditions for captive cephalopods (e.g., providing shelters, intellectual stimulation, a varied environment) is a topic of great interest (Anderson and Wood, 2001; Williams et al., 2009; Baumans and Van Loo, 2013). As with many vertebrates, an enriched environment can positively influence cephalopod behavior as shown in cuttlefishes (Poirier et al., 2004, 2005; Yasumuro and Ikeda, 2016) octopuses (Beigel and Boal, 2006; Yasumuro and Ikeda, 2011), as well as memory formation and animal growth (Dickel et al., 2000). Future studies should test ways of presenting food that stimulate natural foraging behavior and yet are compatible with the ethical treatment of prey species, and identify tank materials and substrates that enable the expression of natural behaviors such as camouflage, hiding and exploration. Of course, environmental enrichment must also always be balanced against the need for good environmental hygiene and the ability to assess the status of the animals (Fiorito et al., 2015).

## Cephalopodan Innovations; Behavioral Plasticity, Advanced Cognition and Sophisticated Neurobiology

Some of the phenotypic features that make cephalopods such atypical invertebrates and so compelling to scientists and casual observers alike include their behavioral plasticity and advanced cognition, supported by sophisticated underlying neural organization. The past decade has seen the publication of a number of excellent reviews and books dealing with these topics singly or in conjunction with each other. For a superb and thorough overview of cephalopod behavior, refer to the recently updated eponymous book by Hanlon and Messenger (2018), as well as reviews by Huffard (2013), Marini et al. (2017), Mather and Dickel (2017) and Villanueva et al. (2017). Body patterning, for the purposes of both signaling and camouflage have been reviewed recently (Borrelli et al., 2006; Tublitz et al., 2006; Mäthger et al., 2009) as for learning and memory capabilities (Borrelli and Fiorito, 2008; Amodio and Fiorito, 2013; Dickel et al., 2013; Mather and Kuba, 2013; Darmaillacq et al., 2014; Tricarico et al., 2014; Zarrella et al., 2015; Mather and Dickel, 2017), while the evolution of cognition in this group is explored in several others (Grasso and Basil, 2009; Godfrey-Smith, 2013, 2016; Vitti, 2013). The mid-20th century brain ablation experiments by J. Z. Young and colleagues are comprehensively surveyed by Sanders (1975), while Marini et al. (2017) offer a briefer and modern synopsis of this work.

Young (1985) summarized early investigations of the visual and equilibrium systems and extraocular photoreceptors, while more recent reviews of sensory systems, particularly vision, are available in a number of works (Budelmann, 1995, 1996; Budelmann et al., 1997; Hanlon and Shashar, 2003; Alves et al., 2008; Nilsson et al., 2012; Dröscher, 2016; Levy and Hochner, 2017; Hanke and Osorio, 2018). Recent developments in the cephalopod neurosciences has been largely based on the initiative of Dr. B. Hochner and colleagues, including study of the cellular, molecular and synaptic mechanisms of the cephalopodan nervous system (e.g., Hochner et al., 2006; Hochner, 2010, 2012, 2013; Zullo and Hochner, 2011; Brown and Piscopo, 2013; Hochner and Shomrat, 2013; Shomrat et al., 2015; Zarrella et al., 2015; Turchetti-Maia et al., 2017).

Because these topics have recently been addressed so extensively, we have opted to focus this section primarily on what we view as pressing near-term challenges and highlight some particularly promising potential methods with which we might investigate them.

### Into the Wild: Studying Cephalopod Behavior

The current understanding of cephalopod behavior is limited by the fact that it has mainly been studied in laboratory settings. Unfortunately, without the ecological context of the natural environment, the survival value *sensu* Tinbergen (1963) of particular behaviors often cannot be perceived, leading to misinterpretations of evolutionary or ecological fitness. Thus, in order to improve understanding of cephalopod behavior, more field observations and field experiments are needed. While there are obvious difficulties to field work, the insight gained will be well-worth the effort. A recent study by Schnell et al. (2015) is a good illustration of this: via controlled laboratory experiments, the authors found that the white lateral stripe displayed by female *Sepia apama* signals non-receptivity for mating (they are less likely to mate when showing it). However, observations of natural behavior in the field showed that males largely ignored this and tried to mate anyway. This combination of laboratory tests with natural observations allowed observers to deduce the intended meaning of an intraspecific signal, but also provided contextual data about its relevance and efficacy in actual mating situations. And where experiments in the field are not possible, we encourage researchers to consider conducting their experiments in the field or in semi-natural conditions (such as a mesocosm), which have the advantage of promoting natural behaviors while also allowing for more experimental control.

The effort to increase the canon of field data will be aided by the pace of technological development and decreasing costs of data acquisition tools. Various types of tagging have been utilized successfully in recent years to answer questions about geographic range, migration and diving habits (Fuentes et al., 2006; Gilly et al., 2006; Replinger and Wood, 2007; Bazzino et al., 2010; Barry et al., 2011; Sims et al., 2011; Wearmouth et al., 2013; Sykes et al., 2017b). Remote monitoring through videography and photography is another increasingly accessible option thanks to the profusion of low-cost cameras that have come on the market in recent years. In particular, the use of cameras mounted onto cephalopods, in or near their dens or on their predators or prey are enabling researchers to study previously inaccessible behavior. For instance, Rosen et al. (2015) were able to use cameras mounted on Humboldt squid to document and analyze two distinct body patterns (“flashing” and “flickering”) *in situ* and to infer their likely purpose as intraspecific signal and dynamic camouflage, respectively. Similarly, remotely-operated underwater vehicles (ROVs), AUVs and submersibles are also becoming more affordable, and have greatly expanded knowledge of deep-sea cephalopod behavior, such as providing evidence as to the purpose of the bizarre asymmetric eyes of cockeyed squids (Thomas et al., 2017) characterizing arm autotomy in a mesopelagic squid (Bush, 2012), and even capturing footage of the elusive giant squid^8^.

In addition to embracing the benefits of this evolving technology, the cephalopod research community should consider sharing this raw video footage and data to an open access repository. Such a repository would allow students and researchers lacking funds, facilities or animals to perform their own analyses and contribute to the body of knowledge. This would be in line with a recent suggestion by other authors (Xavier et al., 2015) who have urged a community-wide shift in focus from data acquisition to data analysis. More importantly, the sharing and reuse of raw data and footage would improve welfare by reducing the total number of animals manipulated for experiments (Fiorito et al., 2014).

Regardless of whether research takes place in the laboratory, mesocosm or the field, greater efforts at standardization across experiments is needed. Due to the sensitivity and advanced perceptive abilities of cephalopods, even minor methodological differences can skew results and lead to divergent conclusions. For example, the standard method of measuring learning and memory in cuttlefish is the “Prawn-in-the-Tube” (PIT) procedure (Messenger, 1973) which has been used for decades by a number of research groups. While this standardized method theoretically allows direct comparisons to be made between experiments conducted in different times and places, the discovery that cuttlefish and other cephalopods are able to perceive differences in the polarization of light has led to the realization that the seemingly irrelevant choice of tube material (i.e., glass versus plastic—each of which alters the properties of light in different ways) could potentially affect results (Cartron et al., 2013). One technique to increase standardization across experiments and research groups is the creation and use of standardized video stimuli (e.g., approach of a predator, prey item or conspecific) from a set of such videos for use in behavioral experiments. Such a system has already been used by one group (Pronk et al., 2010) to study the reactions of octopus over time and between individuals. If such video clips were shared to a common open-access platform as suggested above, experiments could be replicated at different times and by different labs in a standardized fashion using commercially available audiovisual playback equipment.

In addition to standardizing and replicating experiments and observations within the same species, the cephalopod research community should also strive to duplicate across multiple species. Having corresponding data on closely related animals allows comparisons to be made and conclusions to be drawn about the entire lineage by giving a sense of what behaviors are evolutionary conserved from earlier shared ancestors and which represent novel adaptations to the particular environment of that species. In the family Hominidae for example, social differences between such congeners as apes, chimps and bonobos allow assessment of the factors driving behavioral evolution (e.g., Stanford, 1998; Malone et al., 2012). Similar comparisons between such commonly studied cephalopod species as *Sepia officinalis*, *Loligo vulgaris* and/or other squids, and *Octopus vulgaris* would be a good place to start, although the eventual goal should be to assess behavior across a wide variety of species, including the non-coleoid cephalopod *Nautilus* spp. (see for example, Crook and Basil, 2008), which can serve as an ancestral reference point.

Cephalopod research would also benefit greatly from the formal investigation of inter-individual differences and behavioral plasticity in this group. Anecdotal observations by aquarists and researchers give the distinct impression that individual animals have distinct “personalities.” Indeed, in *S. officinalis*, certain behaviors were expressed predictably and consistently over time, although the expression of other behaviors differed between testing situations (see for example findings in Carere et al., 2015). Further research into this subject may indicate different tactics and interpretations need to be applied at the population level, such as distinguishing between “personality types” when calculating group means. Ultimately, plasticity may explain some of cephalopod’s extraordinary evolutionary success, including their evolutionary persistence through three mass extinctions and recent increases in population despite (or perhaps because of) the effects of global climate change as discussed by Doubleday et al. (2016). Behavioral plasticity may buffer cephalopods against the rapid changes in environmental conditions that the world is currently experiencing (e.g., bleached coral reefs, invasive species, changing temperature regimes), and this hypothesis will be put to the test in coming years.

Another anthropogenic environmental impact that is increasingly relevant is how cephalopod behavior is affected by environmental pollutants. As neurologically complex organisms often residing in nearshore environments polluted by pharmaceutical residues, pesticides, and other chemicals, the cephalopod nervous system can potentially be affected. Indeed, the selective serotonin re-uptake inhibiter (SSRI) Fluoxetine, a pharmaceutical product found in high concentrations near heavily populated coastal areas across the globe, has been shown to affect young *S. officinalis* in different ways depending on age and dose (Di Poi et al., 2013; Bidel et al., 2016b). Moreover, in one case, differences could not be identified with standard behavioral tests but only by combining assays (Bidel et al., 2016b), demonstrating that the effects of such pollutants can be subtle and not immediately apparent. Considering the rapid pace of anthropologically induced environmental change, it is important that to get a behavioral “baseline” of vulnerable species as quickly as possible, since such information can be used to guide future environmental and fishing regulations that will mitigate the effects of these pollutants and climatic shifts.

### Alien Intelligence? The Evolution of Advanced Cognition in Cephalopods

Cephalopods demonstrate unexpectedly advanced cognitive abilities and should play a much larger role in scientific discussions about cognitive evolution. A number of cephalopodan features, have experienced convergent evolution with vertebrates, allowing cephalopods to serve as a phylogenetically distant reference point from which to examine the universal selective pressures driving the evolution of organ systems and other traits. For instance, both the vertebrate and cephalopod eye have evolved to function similarly, but via alternative physiological means (review in Fernald, 2000). This demonstrates that despite vast differences in ancestry and underlying physiology, selection can sometimes arrive at the same evolutionary solution to an ecological challenge – in this case, the need to gather highly accurate and detailed visual information from the environment. In a similar manner, cephalopods have enormous potential to reveal the general evolutionary principals driving cognition. By making direct comparisons between cephalopods and “cognitively advanced” vertebrates, such as mammals and birds, the evolutionary pressures driving cognitive evolution, as well as the physiological prerequisites for such advances, can be inferred with less bias from shared ancestry. For instance, the existence of such cognitive abilities as learning and memory in relatively non-social cephalopods demonstrates that sociality is not necessarily a prerequisite for cognitive evolution, and calls the social intelligence hypothesis – the idea that the need to navigate complex intraspecific social interactions may have been the primary driver of cognitive evolution in primates, cetaceans and birds – into question (see Holekamp, 2007). It is also worth mentioning that in a similar manner, cephalopods can also be used as a non-vertebrate model with which to study the nature of animal consciousness (Mather, 2008; Edelman and Seth, 2009; Mather, 2011).

Complex nervous systems and cognition come at a high metabolic cost for organisms (Godfrey-Smith, 2013), and in cephalopods, the size of the brain limits the amount of food that can be ingested per swallow and puts animals at risk of brain injury (Huffard, 2013). Thus, there must be strong selective pressure or pressures (survival value, *sensu* Tinbergen, 1963) driving its evolution in the face of these disadvantages. Cross-phyla comparisons to identify circumstances common to organisms that share this feature are currently underway, and promise fruitful insights in the very near future. Initial comparisons with birds and mammals suggest that a variable environment is an indispensable driver of advanced cognition, since that is a factor common to all three groups (Vitti, 2013), but more investigation is necessary before any concrete conclusions can be drawn. Other potential selective pressures driving cognitive development in this group can be addressed through a better understanding of the timing of evolutionary history in general. For instance, Packard (1972) suggested that cognitive evolution was driven by the rise of and competition with bony fishes, while more recently, other authors (Grasso and Basil, 2009) argue that cephalopodan cognitive development actually occurred long before the advent of bony fishes in response to competition with the first jawed fishes and with other cephalopods. A more comprehensive and precise timeline of evolutionary events during the Paleozoic and Mesozoic will obviously aid in resolving this question.

Like external selective pressures, the proximate mechanistic factors (causation, *sensu* Tinbergen, 1963) that enabled such an impressive degree of cognitive evolution in this group also require investigation. It has been suggested recently that the loss of the hard external shell (Mather, 2011) and the advent of sophisticated vision (Vitti, 2013) were key innovations supporting cognition. However, neural gigantism of the molluscan lineage (Gillette, 1991), than may account for exceptional cerebralization in cephalopods, which increases the transmission efficiency of the molluscan nervous system despite the absence of the vertebrate myelin-sheath gaps, is another factor to consider. The cognitive abilities and behavioral plasticity of cephalopods may also be related to recently discovered dynamic-editing of RNA (Liscovitch-Brauer et al., 2017). Some authors even go so far as to suggest that cephalopod cognition is of alien origin, the result of genes introduced by extraterrestrial viruses that arrived on earth via meteorite 270 million years ago (Steele et al., 2018). To address these hypotheses, a more complete and accurate history of the cephalopod lineage is needed, including more accurate phylogenies as well as more precise timeline of the advent of certain physiological changes and innovations (e.g., shell loss, encephalization). A good first step in this effort would be a more comprehensive survey of the learning abilities of the “living fossil” *Nautilus* (Basil et al., 2011), the extant cephalopod most similar to the putative ancestral condition from which coleoids evolved. Comparisons of the coleoids (150 million years old) with their smaller-brained, less-encephalized *Nautilus* relatives (400 million years old) would allow deduction of the role of various senses and neural structures in the cognitive abilities of cephalopods. The *Nautilus* has only 13 lobes compared to the 40 identified in octopus, and, importantly, lacks a vertical lobe—the structure thought to be the seat of higher cognitive processes in coleoids. Recent experiments with *Nautilus* have demonstrated that they possess more advanced cognitive abilities than traditionally thought, including rapid learning, biphasic memory and advanced olfactory spatial navigation skills (Crook and Basil, 2008; Crook et al., 2009; Basil et al., 2011). This contradicts traditional interpretations of nautilus’ cognition, and suggests that either a prototype vertical lobe system is present in the *Nautilus* (perhaps the plexiform layer and suboesophageal nerve cords), or that the vertical lobe is not as critical to advanced cognition in coleoids as currently thought (sensu Basil et al., 2011).

Inquiries into the cognitive evolution of cephalopods would also be greatly facilitated by increasing the amount of genomic and paleontological data available. For example, comparison of gene expression in the eyes of nautilus, squid, other molluscs and humans has enabled the identification of at least three types of genetic innovations that occurred during evolution of the cephalopod eye, including the duplication and subsequent re-purposing of some genes (Yoshida et al., 2015). Sutton et al. (2016) conducted a phylogenetic analysis on a morphological dataset constructed from both extinct (fossil) and extant specimens, and were able to confirm many of the putative relationships between coleoid groups, but found a few to be para- or polyphyletic. The recent sequencing of the entire *O. bimaculoides* genome has revealed that unlike other molluscs, this species (and probably other octopus species) has experienced expansion of some of the same gene families involved in vertebrate neuronal development (Albertin et al., 2015). Finally, another study used data from 180 genes across 26 species to test hypotheses about divergence times and were able to date the origin of specific groups, including vampire squids, dumbo octopuses, incirrate octopuses and decabrachians (Tanner et al., 2017).

While the recent boom in genetic data has led to some neglect of more traditional paleontological and morphological methods (Xavier et al., 2015), new imaging and phylogenetic techniques are being used to extract more information from existing fossil specimens. For example, UV light has been used to reveal structures not normally visible in a fossilized belemnite (*Acanthoteuthis speciosus*), including cranial cartilage, vague imprints of the statocysts and the first-ever evidence of a belemnitid radula (Klug et al., 2016). Though a fossil record for most soft-bodied cephalopods is lacking, a few specimens do exist. Recently, researchers were able to reconstruct soft body parts in three dimensions (including the eyes and some suckers) from a fossilized octopus using synchrotron microtomography (Kruta et al., 2016). The presence of suckers in this specimen forced researchers to re-evaluate the advent of this structure, which was thought to be a more recent development. Other possible tools include isotope analysis of fossil material and X-ray tomography, a method which allows the internal investigation of fossils and which can reveal preserved soft tissues. Synthesis and integration of information gained from more “traditional” paleontological and phylogenetic methods with data gleaned from modern “omic” tools promises to be a fruitful path forward for the study of cephalopod cognition.

### Action Potential: The Future of Cephalopod Neurobiology

The work conducted by J. Z. Young and colleagues mid-twentieth century continues to serve as the foundation of our understanding of the cephalopod brain and nervous system, and how they control behavior. A lag in progress followed this work (see closing paragraph of Young, 1985), punctuated by a few exploratory experiments (e.g., Bullock and Budelmann, 1991; Williamson and Budelmann, 1991), but interest and improved techniques enabled an uptick in progress starting in the early 2000s. In particular, new neurophysiological approaches were developed in the labs of Drs. B. Hochner (Hebrew University, Israel) and G. Fiorito (Stazione Zoologica Anton Dohrn, Italy) that fueled a resurgence in the study of cephalopod neurophysiology. Electrophysiological recordings from brain-slice preparations in these labs have demonstrated the existence of a long term potentiation similar to that of vertebrates (Hochner et al., 2003) which is considered the cellular analog of long-term memory. A combination of behavioral and electrophysiological approaches have provided insights in the mechanisms involved in short and long-term memory in cephalopods (Shomrat et al., 2008). Comparisons of slice preparations of cuttlefish and octopus show that the vertical lobe of both species although similarly organized express synaptic plasticity in different layers and ‘modes’ (Shomrat et al., 2011), suggesting multiple independent evolutions of this computational system in coleoids. The next step in these electrophysiological efforts will be to adapt the recently developed wireless *in vivo* neural recording techniques (e.g., Hasegawa et al., 2007) to cephalopods, so that brain activity can be monitored as they move freely and perform natural behaviors.

Non-electrophysiological methods have also been recently used to gain insight into the cephalopod nervous system. For instance, anatomical and histological comparisons between the hatchlings of six different coleiod species showed that the sizes and shapes of the visual and nervous systems of various species demonstrate plasticity according to their respective ecological niche (Wild et al., 2015). This information could be useful in situations where the origin of a specimen is unknown—measurement of the relative size of various neural structures might yield clues about its ecological niche, much the same way as tooth shape suggests diet in vertebrates. Another group compared the expression of four genes encoding transcription factors important for nervous system development in squid to that of other bilaterians. They found that the roles of these genes have been largely conserved across these widely divergent groups, and thus represent a shared legacy with other bilaterians (Wollesen et al., 2014).

Further progress in the field depends on the continuing development and adaptation of new neurobiological methods and techniques, and advances in neuroimaging hold particular promise for the study of cephalopod brains. Recently, Bidel et al. (2016a) adapted and validated a method to quantify dopamine, serotonin, norepinephrine and their metabolites simultaneously in brains of cuttlefish using high performance liquid chromatography electro-chemical detection. Array tomography and calcium imaging are two methods which might soon be possible with cephalopods. In array tomography, tissues are stabilized by a glass substrate that allows samples to be stained with multiple markers so that both brain structure and 20 or more neurotransmitters can be viewed simultaneously in three dimensions (Micheva and Smith, 2007). By contrast, neuronal calcium imaging has the advantage that it can be used on animals that are awake and moving (Grienberger and Konnerth, 2012).

As the study of cephalopod neurobiology progresses, it is critical to make every effort to avoid unnecessary pain, suffering, distress and lasting harm (PSDLH) to the animals. This will be greatly facilitated by determining whether or not cephalopods are capable of experiencing pain and suffering, and to validate our standards of anesthesia for this taxon, investigations that are only just beginning (Crook et al., 2013; Alupay et al., 2014; Di Cristina et al., 2015; Butler-Struben et al., 2018). Such work is especially important given recent legislative changes (see above) and our growing knowledge of their sensory and cognitive sophistication. Luckily, technological advances and cost-reductions have made some non-invasive methods available. One example is primary neuronal cell culture, in which neurons are disassociated from the octopus brain and used to establish cell lines that can be cultured and studied *ex vivo* (Maselli et al., 2018), reducing the need for experimentation on live animals. Likewise, ultrasound machines have been used to study brain size in octopus and arm morphology (Grimaldi et al., 2007; Margheri et al., 2011), while non-destructive X-ray microtomography has been used to map the brain of bobtail squid (Kerbl et al., 2013).

As we utilize these methods to glean new data, this and existing information should be digitized and shared as suggested by Xavier et al. (2015), both to facilitate further scientific progress and avoid the unnecessary or redundant use of animals. In particular, the development of online, shared digital brain atlases such as those that exist for rodents (e.g., the Allen Brain Atlas) is within reach and urgently needed for commonly studied cephalopod species such as like *S. officinalis* and *Octopus vulgaris*. Non-digital atlases, already exist for the squids *Sepioteuthis lessoniana* and *I. paradoxus* (Shigeno et al., 2001; Yamamoto et al., 2003), and should be expanded and digitized. Such efforts should include not only physiological structures and gene expression but also extend to mapping the “connectomes” (all of the connections that exist in the nervous system) of the cephalopod brain.

Some important research topics that have been pursued in the last two decades with the various methods described and proposed above are the motor control of posture and limbs, especially regarding the parallel processing necessary to control 8 or 10 appendages of coleoids (e.g., Sumbre et al., 2001, 2005, 2006; Zullo et al., 2009; Levy and Hochner, 2017), as well as neural control of body patterning (Wardill et al., 2012; Rosen and Gilly, 2017) and texture (Gonzalez-Bellido et al., 2018). Finally, the existence and role of sleep in cephalopods, which undergo periods of behavioral and physiological quiescence that strongly resembles sleep in vertebrates (Mather, 2008; Meisel et al., 2011; Frank et al., 2012) is in our view a fascinating area of inquiry that could give insight into the phylogenetic origins and biological reasons for sleep in animals.

## Final Thoughts

In addition to focusing on research and investigation, cephalopod researchers should also be on the lookout for new creative ways to disseminate knowledge and to further augment public awareness and interest. Some novel forms of public outreach that have been used recently include an interactive museum exhibit which encourages visitors to participate in their own neuroscientific data analysis (“Surprising Minds” at the Brighton Sea Life Centre, United Kingdom^9^), a graphic novella illustrating the results of a scientific study (“Cuttlefish Brawl” by Shanna Baker and Mark Garrison^10^) and a virtual reality game allowing visitors to see through the eyes of a cuttlefish (“Eye Sea” by Darmaillacq and Bellanger, 2016^11^). More traditional mediums are important too, of course, and a slew of recent books targeting the non-scientific public (e.g., Williams, 2011; Montgomery, 2015; Godfrey-Smith, 2016; Staaf, 2017) have been published in the last decade.

The public fascination with cephalopods should also be leveraged to promote conservation efforts and to encourage marine research and exploration. Interest could also be channeled in non-traditional ways, such as citizen science via crowd-sourced data collection and analysis. Dozens to hundreds of photographs and videos of cephalopods are shared to social media every year. There is no reason why such media cannot be put to scientific use by posting them to an open access online repository. Aquarists, divers and fishermen should be encouraged to share observations, photographs, videos and data with the cephalopod research community. We could also harness public aid in analyzing large data sets through crowd-sourced analysis, such as the manual assessment of cuttlefish body pattern components or for measuring the size of brain structures from digitized histological thin sections. Public participation is already utilized by marine scientists to collect data (e.g., tag-and-release tracking programs), as well as in analyzing large data sets online (e.g., NASA’s hunt for exoplanets, “Backyard Worlds: Planet 9”^12^, Seabirdwatch^13^). However, it is important to bear in mind that while an animal’s popularity may be harnessed for worthy causes, fame is not without its pitfalls—such as potential overfishing by the hobby aquarium industry, as for the plight of clownfish after the release of *Finding Nemo* (Yan, 2016) or ornamental shell trade (e.g., Nijman and Lee, 2016). Human advocates for cephalopods must work to avoid such exploitation.

Another goal the cephalopod research community should work toward is the development of a shared, open-access platform for data sharing. With a rapidly changing climate and growing food demands, the continued generation and dissemination of data that can guide fisheries and environmental practices is ever more important in order to mitigate human impact. Moreover, it is likely that there are many aspiring cephalopod researchers who may not have access to animals or suitable equipment to conduct their own experiments (e.g., at land-locked academic institutions for instance), but could make use of shared data or media. Shared open-access tools and data can also help pursue cephalopod research in a way which minimizes pain, suffering and lasting harm, by reducing the total number of animals that need to be manipulated and by promoting best-practices. In addition, researchers working in countries where cephalopod research is not currently regulated by animal welfare legislation (i.e., outside of the European Union) or with invertebrate groups that are not currently regulated but will likely be in the future (e.g., bees, decapods), could refer to this platform in developing their own welfare practices. Finally, researchers could use this platform to share information with each other regarding the health and maintenance of animals in their care, and publicize their own research findings. At least two such platforms are currently being developed by the research community: one for the cataloging of cephalopod diseases and parasites for the purpose of improving cephalopod welfare and another for sharing data and media.

Finally, we also feel that it is important to encourage other aspiring cephalopod scientists. Each of the authors was drawn to study cephalopods due to their deep fascination with these animals. Surely other young prospective scientists share this passion, and deserve a productive outlet. The creation of M.Sc. or Ph.D. programs in cephalopod research would be a good first step. Involving early-career researchers in the activities and decisions of the cephalopod scientific community (e.g., conferences, workshops, courses, establishment of welfare guidelines) would also foster and support their development. For those already established in the field, we encourage participation in short courses, training schools and workshops related to cephalopods. Over the past four years (October 2013–September, 2017) the cephalopod community in Europe was able to stage a number of classes, training schools, international meetings and short-term research projects through the support of a COST Action. These have contributed greatly to the standardization of techniques across the field and facilitated networking between labs throughout Europe and beyond. Hopefully, such international exchange will continue, and cephalopod researchers will continue to reach across international borders in order to build interdisciplinary teams that combine different areas of expertise in order to address the challenges discussed here (summarized in **Table 1**).

## Author Contributions

The authors contributed equally to this manuscript, with each writing 3,000–4,000 words. KR composed the sections aquaculture, welfare, and climate change. IW composed the section on genetics. CO composed the General Introduction and Final Thoughts section as well as the sections on cognition, behavior and neuroscience/biology. All authors read and agreed on the final version.

## Conflict of Interest Statement

The authors declare that the research was conducted in the absence of any commercial or financial relationships that could be construed as a potential conflict of interest. The reviewer PS and handling Editor declared their shared affiliation.

## Footnotes

1 2016, Paramount Pictures.

2 2017, Columbia Pictures.

3 2007, Walt Disney Pictures.

4 2016, Walt Disney Pictures.

5 For examples, see YouTube for videos depicting Paul the “psychic” octopus, who “predicted” the results of all of Germany’s World Cup football games in 2010 (https://www.youtube.com/watch?v=kFvrAdyFUJ8, accessed May 3, 2018) or the clip from 2015 of an octopus jumping out of an Australian tide pool to capture a crab (https://www.youtube.com/watch?v=Ar5WJrQik2o, accessed May 3, 2018).

6 https://www.cephseq.org/pioneer-species-list, accessed March 1, 2018

7 www.cephsinaction.org/working-groups/working-group-4/#Database

8 https://www.youtube.com/watch?v=lzrzw4FpoKU, accessed March 1, 2018

9 http://www.everymind.online/SurprisingMinds/, accessed March 1, 2018

10 https://www.hakaimagazine.com/videos-visuals/cuttlefish-brawl/, accessedMarch 1, 2018

11 http://recherche.unicaen.fr/ressources/outils/eye-sea-825922.kjsp, accessedMarch 1, 2018

12 https://www.zooniverse.org/projects/marckuchner/backyard-worlds-planet-9, accessed March 1, 2018

13 https://www.zooniverse.org/projects/penguintom79/seabirdwatch, accessedMarch 1, 2018
